# Why Do Women Reject Bisphosphonates for Osteoporosis? A Videographic Study

**DOI:** 10.1371/journal.pone.0018468

**Published:** 2011-04-13

**Authors:** Elizabeth A. Scoville, Paula Ponce de Leon Lovaton, Nilay D. Shah, Laurie J. Pencille, Victor M. Montori

**Affiliations:** 1 Mayo Medical School, Rochester, Minnesota, United States of America; 2 Universidad Peruana Cayetano Heredia, Lima, Peru; 3 Knowledge and Evaluation Research Unit, Mayo Clinic, Rochester, Minnesota, United States of America; 4 Division of Health Care Policy and Research, Department of Health Sciences Research, Mayo Clinic, Rochester, Minnesota, United States of America; 5 Division of Endocrinology, Diabetes, Metabolism, and Nutrition, Department of Internal Medicine, Mayo Clinic, Rochester, Minnesota, United States of America; 6 Department of Clinical Epidemiology and Biostatistics, McMaster University, Hamilton, Ontario, Canada; Yale University School of Medicine, United States of America

## Abstract

**Background:**

Despite access to effective, safe, and affordable treatment for osteoporosis, at-risk women may choose not to start bisphosphonate therapy. Understanding the reasons women give for rejecting a clinician's offer of treatment during consultations and how clinician's react to these reasons may help clinicians develop more effective strategies for fracture prevention and medication adherence.

**Methods:**

We conducted a videographic evaluation of encounters in the Osteoporosis Choice randomized trial of a decision aid about bisphosphonates vs. usual primary care. Eligible videos involved consultations with women with an estimated 10-year fragility fracture risk >20% who verbalized at least one reason to not take bisphosphonates. Two reviewers independently reviewed eligible videos and verbatim transcripts, classifying patient views about bisphosphonate use, clinicians reponse to those views, and patient adherence at 6 months post visit.

**Results:**

Eighteen video recordings (12 with decision aid) were eligible for analyses. We identified 37 reasons for and against bisphosphonate therapy. Eleven patients rejected treatment, offering 9 (average of 2 per patient) unique reasons against initiating bisphosphonates (most common: side effects 39% and distrust of medications in general 33%). When physicians conceded to patient views the outcome was no bisphosphonate use. Adherence to choices at 6 months was 100%.

**Conclusions:**

The expression of patient preferences is sometimes unfavorable to bisphosphonates treatment even among well-informed patients at high risk for osteoporotic fractures. At 6 months, patients who expressed concerns about these medicines behaved consistently with the decision made during the visit.

## Introduction

Bisphosphonates can reduce the risk of osteoporotic fracture in postmenopausal women [1], [2]. Proper use of these agents is associated with few adverse effects. Generic oral bisphosphonates have recently become available reducing the cost of treatment. Despite access to effective, safe, and affordable treatment, many women at high risk for fractures choose not to initiate therapy, and, of those who do, up to 50% discontinue treatment in less than one year [3]. Limited use of bisphosphonates, whether from poor initiation rates, poor adherence, or high discontinuation, fails to reduce the risk of osteoporotic fractures which in turn can increase healthcare costs and greatly decrease quality of life and life expectancy [4], [5], [6]. Inadequate treatment remains a key quality target in the care of patients with osteoporosis.

To understand why women don't initiate, adhere, or persist with osteoporosis medications, investigators have surveyed and discussed preferences with patients [7], [8], [9]. The most commonly cited reasons to not initiate bisphosphonate treatment include the difficult dosing schedule, fear of adverse events, and patient beliefs that treatment is ineffective and that the condition is not serious enough to merit treatment [10], [11].

To our knowledge, no study has evaluated the reasons patients express to reject a clinician's recommendation of bisphosphonate treatment to prevent osteoporosis fracture during the consultation. Furthermore, there is no study to evaluate whether the nature of these reasons changes when women receive structured individualized information about the efficacy, safety and cost of these medicines during the visit. Also, there is no study to our knowledge to assess how clinicians respond to the rejections of treatment by patients.

To uncover these issues, we sought to determine the reasons women present when expressing hesitation about initiation of bisphosphonates during primary care consultations with clinicians and how these clinicians react by studying video recordings of these consultations. We also sought to determine if these women adhered to their decision at 6 months post visit. We made these recordings of both usual care visits and of visits in which clinicians used a tailored decision aid about bisphosphonates in the context of the Osteoporosis Choice trial (Clinical trials.gov identifier: NCT00578981).

## Methods

### Ethics Statement

The Mayo Clinic Institutional Review Board approved all procedures (IRB ID# 07-003475). Patients and clinicians gave written informed consent for video recording.

### Study Context

This study used video recordings obtained during a randomized trial of a decision aid (Osteoporosis Choice) versus usual primary care in 100 postmenopausal women with osteopenia or osteoporosis by bone mineral density. Osteoporosis Choice is a single-sheet printed decision aid that includes (a) a pictograph showing the patient's estimated risk of a major fragility fracture in 10 years (estimated using the FRAX calculator) [12] and how this risk will be reduced by using bisphosphonates; (b) a list of adverse effects and their likelihood; and (c) an estimation of oral bisphosphonates out-of-pocket costs. Patients were randomly assigned to receive the decision aid or usual care. Trial procedures included video recording of visits to assess how information was shared and discussed in each visit. Patients were recruited from ten academic primary care sites in and within 60 miles of Rochester, Minnesota, United States. Eligible patients were postmenopausal women, age 50 and over with osteopenia or osteoporosis, who were not already taking bisphosphonates or other prescription osteoporosis medications, who their clinicians found eligible for bisphosphonate therapy and had a follow-up appointment with that clinician. Women who could not read English or had, in their clinicians' judgment, major learning barriers were excluded from this trial. Details of the methods and main trial results are available elsewhere [13], [14]. The decision aid was found to significantly improve patient knowledge and involvement in clinical decision making and to have minimal if any impact on medication adherence. The Mayo Clinic Foundation for Medical Education and Research funded this trial. The funding source had no role in the design, conduct, or decision to publish results of this trial.

### Video selection

Two investigators, working independently, reviewed all available videos from the Osteoporosis Choice trial. Eligible videos for inclusion in this study were (a) of visits with women at FRAX-estimated 10-year fragility fracture risk >20%; (b) receiving either decision aid or usual care; (c) in which the patient verbally expressed at least one reason against treatment with bisphosphonate; (d) regardless of whether the final decision made was to accept or reject treatment. There were no exclusion criteria.

### Data collection

The trial database provided background information about the patients and clinicians. A professional medical transcriptionist transcribed the audio channel of the selected videos verbatim.

Two reviewers (ES and PL), working independently, watched the video recordings and read transcripts for each encounter. Each reviewer recorded verbatim every reason a patient verbalized for or against bisphosphonate treatment.

Patients and clinicians reported on the decision to use or not to use bisphosphonates immediately after the visit; patients reported ongoing use of bisphosphonates at 6 months post index visit. We obtained and reviewed pharmacy medication profiles obtained during the original trial for each patient at 6 months post index visit.

### Data analyses

Before data collection, using the available literature and discussion among study team members, we determined seven categories in which to classify patient's reasons offered for using or not using bisphosphonates. These categories were against treatment (a) side effects, (b) distrust of medications, (c) patient knowledge against treatment, (d) low value of potential benefits, (e) cost of medication, and in favor of treatment (a) high value of benefits, (b) patient knowledge in favor of treatment.

We also noted how clinician's responded to the reasons patients offered, i.e., by agreeing or disagreeing with the patient or by presenting new information; and how patients in turn responded, i.e., by agreeing or disagreeing and pushing for further deliberation. Based on these observations, we described each discussion as (a) give-and-take deliberation, (b) physician dominated, or (c) patient dominated. Deliberation took place when clinicians engaged the patient in conversation by presenting new information in response to patient's concerns and expressing agreement or disagreement with patient views as appropriate. Patient-dominated responses were those where the clinician accepted the patient's opinion regardless of patient's reasoning and did not present new information that would contradict patient views. Clinician-dominated conversations were those where the clinician insisted on his or her expressed opinion while presenting little or no new information that would support the patient's views.

After identifying and categorizing patient and clinician views, utterances, and reactions, the reviewers met to compare notes and discuss disagreements.

## Results

**Figure 1:**
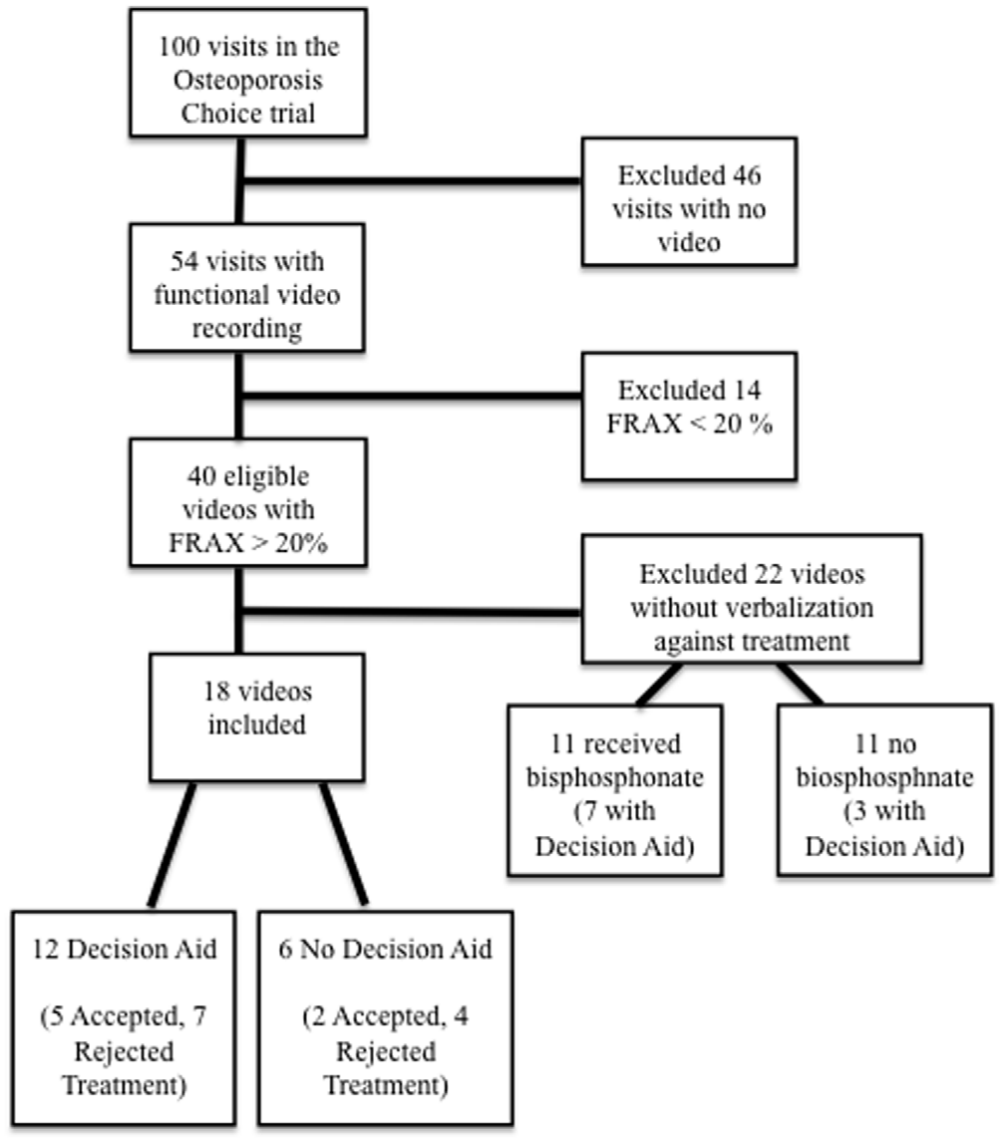
Flow diagram for video inclusion.

Only 76 of the 100 enrolled patient visits were video recorded. Of the high-risk women, 22 had videos in which women made no statement against using these medicines, leaving 18 eligible videos (**Figure 1**). **Table 1** summarizes the characteristics of patients and providers participating in this analysis. Seventeen of the visits were with physicians and one with a nurse practitioner.

**Table 1:** Clinician and patient characteristics.

	All encounters (N = 18)	Accepted treatment (N = 7)	Rejected treatment (N = 11)
**Clinicians**			
Age, mean (SD)	42.7 (10.3)	38.1 (10.2)	45.5 (8.2)
Male, n (%)	12 (67)	3 (43)	9 (82)
Internal Medicine, n (%)	14 (77.8)	6 (85.7)	8 (72.7)
Family Medicine, n (%)	4 (22.2)	1 (24.3)	3 (27.3)
**Patients**			
Age, mean (SD)	70.6 (9.4)	71.7 (25.3)	70.0 (12.3)
Prior fracture, n (%)	12 (67)	5 (71)	7 (64)
Prior bisphosphonate use, n (%)	1 (5)	0 (0)	1 (9)
Estimated 10-year probability of major fragility fractures, mean (SD)	33.8 (18.8)	41.4 (25.3)	28.9 (12.3)
Percentage correct answers on bisphosphonate knowledge, mean (SD)	50.6 (27.0)	62.6 (22.4)	43.1 (30.4)
Identified own 10-year risk of fracture, n (%)	10 (55.6)	3 (43.0)	7 (63.6)
Identified absolute risk reduction in fracture risk with treatment, n (%)	6 (33.3)	2 (29.0)	4 (36.4)

### Reviewer agreement

We identified 37 reasons for and against bisphosphonate therapy: both reviewers independently identified the same 29 (78%) reasons; one reviewer identified 8 additional reasons that were missed by the other and that – upon discussion and agreement—were all included for analysis. Four (14%) of the 29 reasons recorded by both reviewers were classified in different categories; these disagreements were resolved by discussion and consensus.

### Patient views

**Figure 2:**
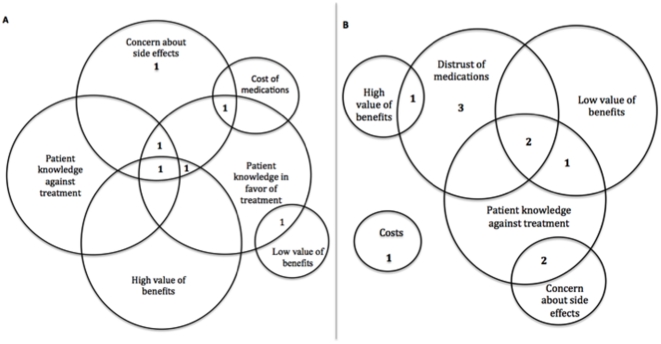
Reasons to take or not take bisphosphonates. Most women gave multiple reasons for and against bisphosphonate therapy. This figure represents the overlap of multiple reasons presented during a single visit for patients who A) accepted treatment and B) rejected therapy.

Seven patients accepted and 11 rejected treatment. Patients offered 4 unique reasons for and 9 unique reasons against starting bisphosphonates (**Table 2**). On average, each patient offered two reasons for or against therapy regardless of whether they ultimately accepted or rejected treatment. **Figure 2** demonstrates the complexity of these conversations by illustrating the overlapping nature of patient reasons. Ten of the 11 patients who refused treatment did not express any positive views about using bisphosphonates. The use of the decision aid did not appear to alter patients' verbalizations of reasons for or against therapy or the patient's final decision.

**Table 2:** Frequency of patient verbalizations for and against bisphosphonate treatment as well as physician response style in relation to acceptance or rejection of treatment.

	Total, n (%)	Accept treatment, n (%)	Reject treatment, n (%)	Representative quote
**A. Verbalized against treatment**				
1. Concern about side effects	7 (39)	5 (71)	2 (18)	“The jaw thing frightens me.”
2. Distrust of medications	6 (33)	0 (0)	6 (55)	“I won't take pills so don't ask.”
3. Patient knowledge against treatment				
a. Family member with no osteoporosis complication	3 (17)	0 (0)	3 (27)	“My mother was 96 before she broke a bone.”
b. History of adverse effect (personal or other)	3 (17)	2 (29)	1 (9)	“I think my mother took this and it made her legs and feet swell”
c. Health good without other treatments	1 (6)	0 (0)	1 (9)	“In general my health's pretty darn good overall, so why mess with a good thing?”
4. Low value of potential benefits				
a. Too old to benefit	3 (17)	1 (14)	2 (18)	“I don't want to live that long”
b. Limited knowledge of osteoporosis	2 (11)	0 (0)	2 (18)	“If I felt bad…[I would consider treatment]”
c. Medications will not produce benefit	2 (11)	1 (14)	1 (9)	“It won't make it get better?”
5. Cost of medication	2 (11)	1 (14)	1 (9)	“If it's not too expensive.”
**B. Verbalized in favor of treatment**				
1. High value of benefits	3 (17)	2 (29)	1 (9)	“Ok, because I don't want to go back to a nursing home”
2. Patient knowledge in favor of treatment				
a. Family member with poor outcome	3 (17)	3 (43)	0 (0)	“My mother fell and broke her hip. That was the end of it”
b. Personal research and insight	2 (11)	2 (29)	0 (0)	
**C. Conversation Style**				
Patient dominated	4 (22)	0 (0)	4 (36)	
Patient-clinician deliberation	14 (78)	7 (100)	7 (64)	
Physician dominated	0 (0)	0 (0)	0 (0)	

### Clinician response

Most clinicians engaged patients in deliberation about the option to treat (**Table 2**). All patient-dominated interactions resulted in the patient not initiating treatment; there were no physician-dominated interactions. Patients who expressed a distrust of all medications in the course of discussion were more likely to elicit a patient dominated response from their physicians (4 out of 6 interactions with a patient who expressed generic distrust of medications were patient dominated).

### Deferred response to physician

Three of the four patients who deferred the decision to the physician or asked the physician for advice ultimately took the advice. Of these, two physicians recommended starting medication and two recommended delaying bisphosphonate treatment. Both patients for whom physicians recommended delaying treatment chose not to initiate treatment (both were in the decision aid group). Of the two physicians who recommended delaying treatment one wished to review records of a previous bone density but a chart review determined that the physician never follow up with the patient, while the other physician did not think the patient warranted treatment at this time and suggested the patient follow up with a repeat bone scan in one year.

### Decision aid vs. usual care

Patients decided to take treatment in five (42%) discussions in which the decision aid was used and in two discussion in which it was not used. The conversations were grossly similar between the two groups. (**Table 3**) Notably, all 3 discussions in which patients expressed placing a high value on the benefits of treatment occurred in the decision aid group, with none occurring in the usual care group.

**Table 3:** Frequency of patient verbalizations for and against bisphosphonate treatment as well as physician response style in relation to decision aid or control group.

	Decision Aid, n (%)	Control, n (%)
**A. Verbalized Against Treatment**		
1. Concern about side effects	5(42)	2(33)
2. Distrust of medications	3(25)	3(50)
3. Patient knowledge against treatment	5(42)	2(33)
4. Low value of potential benefits	5(42)	2(33)
5. Cost of medication	1(8)	1(17)
**B. Verbalized Pro Treatment**		
1. High value of benefits	3(25)	0(0)
2. Patient knowledge in favor of treatment	4(33)	1(17)
**Conversation Style**		
Patient dominated	1(8)	3(50)
Patient-Clinician deliberation	11(92)	3(50)
Physician dominated	0(0)	0(0)

### 6-month follow up

No patients in this substudy revised their decision within 6 months of the initial consultation. That is, all patients who started therapy remained on therapy (with perfect adherence as judged by pharmacy profile review) and no patients who initially declined therapy started taking bisphosphonates in this period.

## Discussion

### Our findings

We found high-risk patients who expressed treatment preferences to their clinician that were unfavorable to bisphosphonate therapy. Patients with only such preferences were more likely to leave the visit rejecting therapy than those who expressed at least one reason to take treatment. The most common reasons expressed were a concern about side effects (39%) or a distrust of medications in general (33%). Patients included in this analysis, all of whom were able to express a reason against bisphosphonates and discuss it with their clinician, adhered to their decision (to take or not to take bisphosphonates) for at least 6 months post visit.

### Limitations and strengths of this study

Few eligible videos – i.e., videos of women at high risk for fracture stating at least one reason to not take treatment – were available for review. Although small, our sample was representative of the whole of the Osteoporosis Choice trial population in age, socioeconomic status, and previous medication use. Our sample has a higher rate of previous fracture and 10-year risk of major fragility fracture as we purposefully selected high-risk women. While fit to our purpose of identifying expressions that factor into patient's decision to reject bisphosphonate therapy, this study does not offer the depth of more complex communication analyses or qualitative attempts to identify the underlying framework of treatment refusal. Also, we did not include videos of women expressing nonverbally their preference for osteoporosis treatment.

Due to the difficulty of blinding reviewers to the outcome of the encounter as it was often expressed through the course of the video recording, we tried to limit bias by having duplicate independent review. While we had a 78% initial agreement and 100% agreement by consensus, this may not have been sufficient to prevent error in categorization. Our videographic analysis looked at only one discussion of bisphosphonate therapy. While none of our patients altered their treatment through the course of 6 months of follow up, it is impossible to know how many of these decisions may have been revisited after that period. The population studied had adequate access to primary and specialty care, and to prescription drug coverage. Therefore, their preferences may not apply to women with limited health care access and literacy.

This study also has some strengths. Participants were unaware of the goal of the sub-study; we did not rely on post visit recall to determine the reasons women had for taking or not bisphosphonates; two reviewers working independently assessed the encounters using pre-defined categories; and for at least 12 of the 18 videos, the preferences expressed should be considered informed as these patients had just reviewed an effective decision aid with their clinician.

### Comparison with other studies

Many of the reasons that women verbalized to their clinicians for not desiring bisphosphonate treatment are similar to those elicited in surveys including a distrust of all medications and concerns of side effects [10], [11]. Prior investigations used surveys not directly linked to a particular visit, and usual care visits seldom succeed in informing women about the pros and cons of therapies in a tailored way. To this extent, our study overcomes these limitations and further indicates that these patients sometimes express these preferences directly to their clinicians.

### Implications for practice, policy, and further research

Policy discussions have increasingly endorsed the notion that shared-decision making can improve the appropriateness and efficiency of health care delivery. This videographic evaluation suggests that shared-decision making may provide a means by which to identify women at high risk of osteoporotic fractures whose preferences may not align with evidence-based practice guidelines. By identifying these women, clinicians may be able to tailor regimens to align with their preferences, thus, reducing non-adherence (to programs women do not want to follow) and improving healthcare value. Although our study does not provide direct evidence of this, the kind of reasons women in this substudy offered would logically reject a common solution proposed to the problem of nonadherence to weekly oral bisphosohonates: the use of less frequently administered oral or intravenous bisphosphonates.

By virtue of their rejection of bisphosphonates, women may force practitioners to become more proficient at recommending nonpharmacological means of reducing the burden of osteoporotic fractures in these high risk women. Such regimens may include measures to improve posture, fitness and balance, reduce visual, mechanical and propioceptual limitations, and prevent falls. The extent of this proficiency and of the necessary resources to support the implementation of these regimens remains unclear.

### Conclusions

The expression of patient values to the clinician is sometimes unfavorable to bisphosphonate therapy even in women at high risk of osteoporotic fracture. Patients who express only values unfavorable to bisphosphonate therapy are more likely to leave the visit rejecting therapy than those who express at least one value favorable to treatment. Patients who discussed at least one concern about these medicines were adherent to their decision to take or not bisphosphonates 6 months post visit. Patient-centered osteoporosis care to reach at-risk women may require a change in priority from drug programs (e.g., changing formulation, generics) to nonpharmacological strategies (e.g., fall prevention).
